# Development of an optimized and practical pharmacokinetics/pharmacodynamics analysis method for aztreonam/nacubactam against carbapenemase-producing *K. pneumoniae*

**DOI:** 10.1093/jac/dkad033

**Published:** 2023-02-13

**Authors:** Yuki Igarashi, Wataru Takemura, Xiaoxi Liu, Nana Kojima, Takumi Morita, Victor Tuan Giam Chuang, Yuki Enoki, Kazuaki Taguchi, Kazuaki Matsumoto

**Affiliations:** Division of Pharmacodynamics, Keio University Faculty of Pharmacy, 1-5-30 Shibakoen, Minato-ku, Tokyo 105-8512, Japan; Division of Pharmacodynamics, Keio University Faculty of Pharmacy, 1-5-30 Shibakoen, Minato-ku, Tokyo 105-8512, Japan; Division of Pharmacodynamics, Keio University Faculty of Pharmacy, 1-5-30 Shibakoen, Minato-ku, Tokyo 105-8512, Japan; Division of Pharmacodynamics, Keio University Faculty of Pharmacy, 1-5-30 Shibakoen, Minato-ku, Tokyo 105-8512, Japan; Division of Pharmacodynamics, Keio University Faculty of Pharmacy, 1-5-30 Shibakoen, Minato-ku, Tokyo 105-8512, Japan; Discipline of Pharmacy, Curtin Medical School, Faculty of Health Sciences, Curtin University, GPO Box U1987, Perth, Western Australia 6845, Australia; Division of Pharmacodynamics, Keio University Faculty of Pharmacy, 1-5-30 Shibakoen, Minato-ku, Tokyo 105-8512, Japan; Division of Pharmacodynamics, Keio University Faculty of Pharmacy, 1-5-30 Shibakoen, Minato-ku, Tokyo 105-8512, Japan; Division of Pharmacodynamics, Keio University Faculty of Pharmacy, 1-5-30 Shibakoen, Minato-ku, Tokyo 105-8512, Japan

## Abstract

**Background:**

Nacubactam, a new β-lactamase inhibitor with antibacterial activity, is being developed as a single drug to be co-administered with cefepime or aztreonam. However, determining pharmacokinetics/pharmacodynamics (PK/PD) parameters in β-lactam/β-lactamase inhibitor combinations remains challenging. We aimed to establish a practical PK/PD analysis method for aztreonam/nacubactam that incorporates instantaneous MIC (MIC_i_).

**Methods:**

Based on chequerboard MIC measurements, MIC_i_ of aztreonam against carbapenemase-producing *Klebsiella pneumoniae* in the presence of nacubactam was simulated.

**Results:**

The mean change in the bacterial count of thigh-infected mice in an *in vivo* PD study was plotted based on %*fT*_>MICi_ and analysed using the inhibitory effect sigmoid *I*_max_ model. *fT*_>MICi_ calculated from the PK experiments showed a high correlation with the *in vivo* bactericidal effect, suggesting that *fT*_>MICi_ is the optimal PK/PD parameter for aztreonam/nacubactam. The target values of *fT*_>MICi_ achieving growth inhibition, 1 log_10_ kill and 2 log_10_ kill, were 22, 38% and 75%, respectively.

**Conclusions:**

The PK/PD analysis method proposed in this study is promising for determining practical PK/PD parameters in combination therapy. In addition, this is the first report of aztreonam/nacubactam showing a potent *in vivo* therapeutic effect against NDM-producing *K. pneumoniae*.

## Introduction

Since the late 1900s, the development of many antimicrobial agents has greatly improved human health and welfare. However, the increase in drug-resistant bacteria due to the inappropriate use of antimicrobials has become a problem worldwide. Recently, the Antimicrobial Resistance Collaborators^1^ reported that in 2019, 4.95 million deaths were associated with drug-resistant bacteria, of which 1.27 million were directly attributable to drug-resistant bacteria, strongly indicating that overcoming drug resistance is an important global healthcare challenge. The O’Neill report^2,3^ estimates that 10 million people will die annually from drug-resistant bacterial infections in 2050 if drug resistance increases at its current rate. In particular, Gram-negative bacteria that have acquired resistance by producing β-lactamases that hydrolyse β-lactam antibiotics are positioned as a serious threat.^4^ To treat patients with β-lactamase-producing Gram-negative bacterial infections, the combination therapy of β-lactams and β-lactamase inhibitors, which inhibit the enzymatic inactivation of β-lactams by β-lactamases, has been the cornerstone of treatment of β-lactamase-producing Gram-negative bacterial infections in modern medical care.

In the clinical use of antimicrobials, pharmacokinetics/pharmacodynamics (PK/PD) parameters calculated using mouse infection models have contributed to determining evidence-based clinical doses of antimicrobials.^5,6,7^ In general, it is desirable to set the PK/PD parameters using specific MICs and pharmacokinetics parameters that can directly reflect the antimicrobial dose to be generalized to a wide variety of bacteria and doses. The PK/PD parameters for most antimicrobial monotherapies in clinical use are based on the percentage of free time above MIC (%*fT*_>MIC_) or *f*AUC/MIC and free maximum concentration (*fC*_max_)/MIC to set the clinical dose.^7^

In contrast, the PK/PD parameters proposed for β-lactam/β-lactamase inhibitors are limited to *fT*>*C*_T_.^8^ The *C*_T_ value, meaning ‘the lowest concentration of β-lactamase inhibitor required to inhibit β-lactamase when used with a given dose of β-lactam’,^9^ is based on the fixed values of factors such as MIC of the bacterial strain, β-lactam administration method, β-lactamase genotype and gene expression levels. This makes it impossible to analyse drug efficacy with flexibility for bacteria with different MICs and concentrations of both drugs using *fT*>*C*_T_, thus preventing comprehensive clinical efficacy prediction. It is difficult to determine the PK/PD parameter that reflects the instantaneous variation of MICs in the presence of fluctuating blood concentrations of β-lactams/β-lactamase inhibitors over time, given the interdependency in the efficacy of the β-lactams/β-lactamase inhibitors. Therefore, PK/PD parameters for β-lactam/β-lactamase inhibitors using *fT*>*C*_T_ values are not practical. Hence, the establishment of new PK/PD parameters incorporating the concept of MIC is desired.

We considered that using the instantaneous MIC (MIC_i_) for PK/PD analysis of β-lactams/β-lactamase inhibitors could overcome this challenge. MIC_i_ is a mathematical modelling and simulation assessment concept that depends on β-lactamase inhibitor concentration and the sensitivity for β-lactams that varies over time. This concept was first proposed by Bhagunde *et al.*^10^ in the imipenem/relebactam *in vitro* hollow-fibre infection model. The utility of MIC_i_ has since been demonstrated for several β-lactams/β-lactamase inhibitors.^11–14^ Therefore, applying the MIC_i_ concept to PK/PD analysis in a murine infection model allows for flexible clinical dosing design, considering the interdependence of β-lactams/β-lactamase inhibitors. However, no *in vivo* studies have proven the value of PK/PD analyses with MIC_i_.

Recently, nacubactam (OP0595), a diazabicyclooctane (DBO)-type new β-lactamase inhibitor with antibacterial activity, is being developed as a single drug to be co-administered with cefepime or aztreonam. Our study aims to establish a PK/PD analysis approach for β-lactam/β-lactamase inhibitors using MIC_i_ and investigate their superiority to *fT*>*C*_T_. For this purpose, we used nacubactam, a novel β-lactamase inhibitor currently under development, and aztreonam, a β-lactam antibiotic. Furthermore, we used the PK/PD analysis method established in this study to explore the practical PK/PD parameters, and their target values of aztreonam/nacubactam against β-lactamase-producing Gram-negative bacteria.

## Materials and methods

### Antimicrobials

Nacubactam monohydrate was provided by Meiji Seika Pharma Co., Ltd. (Tokyo, Japan). Aztreonam powder (Tokyo Chemical Industry Co., Ltd., Tokyo, Japan) and aztreonam vials (Eisai Co., Ltd., Tokyo, Japan) were used for *in vitro* and *in vivo* studies, respectively.

### Bacterial strains

*Escherichia coli* ATCC^®^ 25922 was used as the quality control strain for susceptibility testing. Three strains of β-lactamase-producing *Klebsiella pneumoniae* listed in Table 1 were used in this study. *E. coli* ATCC 25922 and *K. pneumoniae* ATCC^®^ BAA-2473 were obtained from ATCC^®^. Clinical isolates (*K. pneumoniae* MSC 21664 and MSC 21444) were provided by Meiji Seika Pharma.

**Table 1.** Characteristics of the strains used

		MIC (mg/L)^a^		
Strain	β-Lactamase	Aztreonam	Nacubactam	Aztreonam/nacubactam^b^
*K. pneumoniae*				
*ȃ*ATCC BAA-2473	NDM-1	256	256	1
*ȃ*MSC 21664	IMP-6, CTX-M-2	32	16	0.016
*ȃ*MSC 21444	OXA-48	512	2	1

MIC value is the median of technical replicates (*n* = 3).

MIC of nacubactam measured with a fixed aztreonam concentration of 4 mg/L.

### Chequerboard assay

According to CLSI guidelines,^15,16^ chequerboard MICs of aztreonam/nacubactam were determined by broth microdilution using CAMHB (Becton, Dickinson and Company, Franklin Lakes, NJ, USA) (*n* = 3). A 2-fold step dilution series of each drug in the concentration range of 0 and 0.001 to 1024 mg/L was added to the wells of a 96-well plate in a 1/1 volume of aztreonam/nacubactam. Bacteria were added to the plate to a final concentration of approximately 2–8 × 10^5^ cfu/mL. After incubating at 37°C for 20 h, the lowest concentration of aztreonam with each nacubactam concentration, at which no bacterial growth was observed, was defined as the MIC. The MIC values for each drug alone and nacubactam when combined with 4 mg/L of aztreonam are shown in Table 1. MIC values were taken as the median of technical replicates (*n* = 3).

### Effect model analysis

The results obtained by chequerboard assay for each strain were plotted and analysed using the inhibitory effect sigmoid *I*_max_ model (Equation 1) to draw an approximate curve representing the dose–response relationship of the aztreonam MIC to nacubactam concentration.


(1) $$log_{2}(MIC)=log_{2}(MIC_{0})-(I_{max}\times C_{NAC}{}^{\gamma })/(C_{NAC}{}^{\gamma }+IC_{50}{}^{\gamma })$$

where MIC_0_ is the MIC of aztreonam alone, *C*_NAC_ is the concentration of nacubactam (mg/L), *I*_max_ is the maximum inhibitory effect, IC_50_ is the concentration of nacubactam at which 50% of the maximum inhibition occurs and ɤ is the sigmoid coefficient.

### Animals

Male ICR mice (Sankyo Labo Service Corporation, Inc., Shizuoka, Japan) were used for all animal experiments. The mice were acclimatized for 1 week under a 12 h light/dark cycle with feeding and watering *ad libitum* and used for the experiment at 5 weeks of age. The mice were intraperitoneally injected with cyclophosphamide (Shionogi & Co., Ltd., Osaka, Japan) for 4 days (150 mg/kg) and the day (100 mg/kg) before the experiment to decrease their neutrophils. Protocols for all animal experiments were approved by the Institutional Animal Care and Use Committee of Keio University (No. 19037) and the Animal Experiment Management Committee, Pharmaceutical Research Center, Meiji Seika Pharma.

### PK analysis

Aztreonam/nacubactam was subcutaneously administered to neutropenic mice. Blood samples were collected through cardiac puncture under anaesthesia at 5, 15, 30, 60, 120, 240 and 300 min after administration (*n* = 3 per timepoint). Immediately after blood sampling, the mice were euthanized by cervical dislocation. The blood samples were centrifuged to separate the plasma and stored at −80°C. PK parameters were calculated by compartmental model analysis of free plasma nacubactam and aztreonam concentrations using Phoenix^®^ WinNonlin^TM^ (v. 8.0, Certara, NJ, USA).

### LC-MS/MS analysis

The concentration of antibiotics in each plasma sample was measured using a validated LC-MS/MS method. Detailed measurement conditions are summarized in Table S1. The protein binding rates of nacubactam and aztreonam in mouse plasma were determined by the ultrafiltration method. In short, plasma containing nacubactam and aztreonam at concentrations of 1, 10 and 400 mg/L (*n* = 3 per concentration) was incubated at 37°C for 30 min and then set into Centrifree^®^ 4104 Ultrafiltration Centrifugal Filters (Millipore, Bedford, MA, USA). The filtrate was obtained by centrifugation at 1500× g for 5 min. The concentrations of the antibiotics in the initial sample and the filtrate were measured using LC-MS/MS, and the protein binding rate was calculated.

### PD study

Neutropenic mice were infected intramuscularly with 100 µL of inoculum bacterial suspension at a concentration of 1 × 10^6–7^ cfu/mL in the left thigh 2 h before drug administration. A combination of nacubactam (0, 1.2, 3.6, 12, 36, 120, 360, 1200 mg/kg/day) and aztreonam (0, 1200, 2400, 4800 mg/kg/day) were subcutaneously administered from 2 to 26 h after inoculation (*n* = 3 per dose). In the dose-fractionation study, aztreonam was administered q2h. In contrast, nacubactam was administered at a variable interval schedule (q2h, q4h, and q8h) from 2 to 26 h after inoculation (Figure S1). In the dose-ranging study, both drugs were administered q2h from 2 to 26 h after inoculation (Figure S1). The control mice were euthanized by cervical dislocation at 0 h (2 h after inoculation). Other mice were euthanized 24 h after initial drug dosing (26 h after inoculation). The thigh was then aseptically collected and homogenized using Multi-Beads Shocker (Yasui Kikai Corp., Osaka, Japan). Serial dilution series of each homogenate were prepared, and an aliquot of each suspension was applied to Mueller–Hinton agar plates. After incubation at 37°C for about 20 h, the number of colonies that grew was measured. The lowest detection limit was 2.20 log_10_ cfu/thigh.

### PK/PD analysis with fT_>MICi_

**Figure 1.**
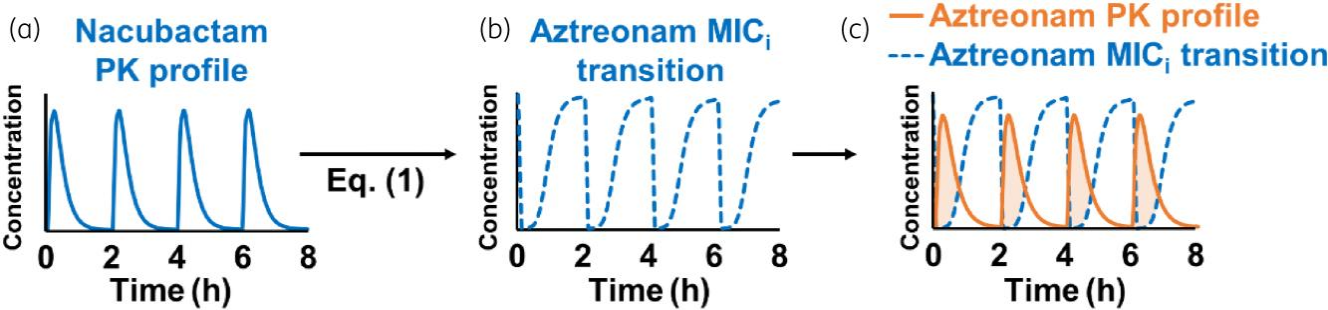
Estimation of *fT*_>MICi_. (a) Simulated free plasma nacubactam PK profile; (b) calculated aztreonam MIC_i_ transition after administration of nacubactam based on the dose–response curve; (c) free plasma aztreonam PK profile (solid orange line) and aztreonam MIC_i_ transition (blue dashed line) were superimposed. The %*fT*_>MICi_ is the percentage of time that the free plasma aztreonam concentration exceeded the MIC_i_. This figure appears in colour in the online version of *JAC* and in black and white in the print version of *JAC*.

The dose–response curve (Equation 1) obtained by the model analysis of the chequerboard assay represents the MIC of aztreonam, which varies depending on the nacubactam concentration. Since the nacubactam concentration is constantly changing *in vivo*, the MIC of aztreonam is also constantly changing accordingly. This instantaneous MIC of aztreonam was taken as MIC_i_. Therefore, by applying the time course of free nacubactam plasma concentration (Figure 1a) to the dose–response relational equation Equation 1, the time course of aztreonam MIC_i_ following nacubactam administration was calculated (Figure 1b). The percentage of time that the free aztreonam plasma concentration exceeded the MIC_i_ was defined as %*fT*_>MICi_ (Figure 1c). The mean change in the bacterial count of each group obtained by *in vivo* PD study was plotted based on %*fT*_>MICi_ and analysed using the inhibitory effect sigmoid *I*_max_ model (Equation 2).


(2) $$E=E_{0}\text{–}I_{max}\times (fT>MIC_{i}{)}^{\gamma }/\{(fT>MIC_{i}{)}^{\gamma }+IC_{50}{}^{\gamma }\}$$

where *E* is the change in the bacterial count, *E*_0_ is bacterial count at *fT*_>MICi _= 0%, *I*_max_ is the maximum inhibitory effect, IC_50_ is the *fT*_>MICi_ at which 50% of the maximum inhibition occurs and *ɤ* is the sigmoid coefficient.

### PK/PD analysis with C_T_

*fT*>*C*_T_, *f*AUC and *fC*_max_ of β-lactamase inhibitors were used as PK/PD indices.^17–19^ Following their lead, the results of the *in vivo* PD study against *fT*>*C*_T_, *f*AUC and *fC*_max_ of nacubactam were analysed. The percentage of time that the free nacubactam plasma concentration at each dose exceeded the *C*_T_ value (0.125, 0.25, 0.5, 1, 2 and 2.5 mg/L) was calculated as %*fT*>*C*_T_. The *C*_T_ value was set with reference to past reports.^17,18,19^ The mean change in bacterial counts of each group obtained from the *in vivo* PD study was plotted based on % *fT*>*C*_T_ and analysed using Equation 2 for each strain and dose of aztreonam used in combination to determine the *C*_T_ value with the highest *R*^2^ value.

## Results

### Chequerboard MIC and effect model analysis

**Figure 2.**
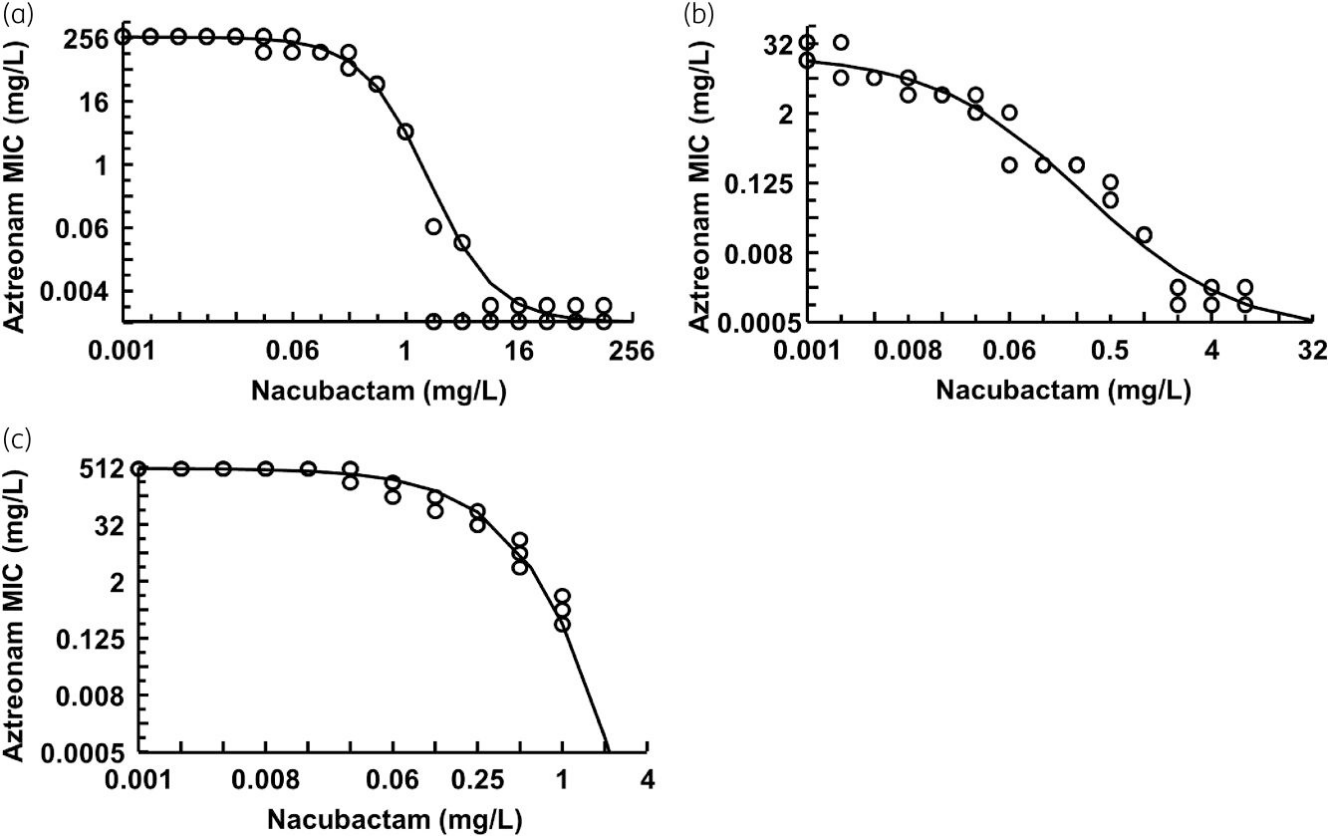
MIC curve between the aztreonam MIC and the nacubactam concentration (*n* = 3). (a) NDM-1-positive *K. pneumoniae* ATCC BAA-2473; (b) IMP-6-positive *K. pneumoniae* MSC 21664; (c) OXA-48-positive *K. pneumoniae* MSC 21444.

The β-lactamase genotype of each bacterial strain and the MIC values of aztreonam/nacubactam alone and nacubactam in the presence of 4 mg/L aztreonam for each bacterial strain are shown in Table 1. Figure 2 shows the dose–response relationship of aztreonam MIC to nacubactam concentration for each strain analysed by Equation 1. All strains were resistant to aztreonam alone, but the combination of aztreonam and nacubactam decreased MIC in a nacubactam concentration-dependent manner. Interestingly, nacubactam alone also showed slight antibacterial activity against all bacterial strains.

### PK analysis

**Figure 3.**
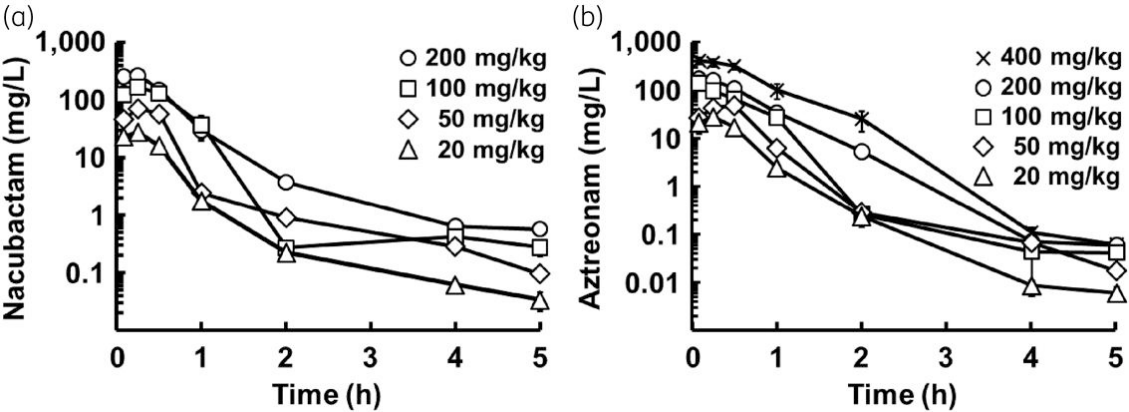
PK profiles of nacubactam (a) and aztreonam (b) in neutropenic mice (mean ± SD, *n* = 3).

The PK profiles of nacubactam and aztreonam in neutropenic mice are shown in Figure 3. The plasma concentration curve for nacubactam and aztreonam best fitted the two-compartment model. The PK parameters of each drug analysed from the plasma concentration curve are listed in Table S2. *C*_max_ and AUC of each drug showed good linearity within the dose range tested (Figure S2). The plasma protein binding rates of nacubactam and aztreonam were similar among the three concentrations (Table S3), with averages of 3.87% and 57.1% for nacubactam and aztreonam, respectively. The average values were used for PK/PD analysis.

### PD study

**Figure 4.**
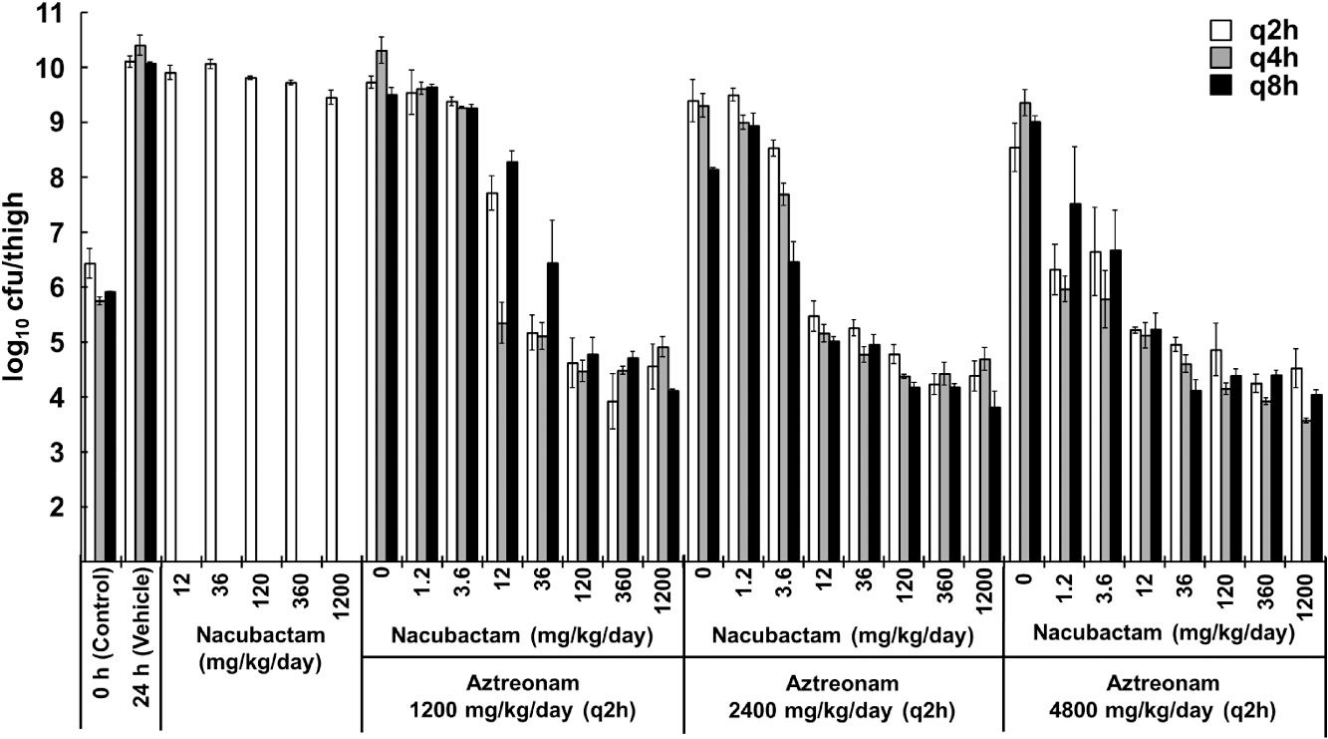
*In vivo* dose-fractionation study of aztreonam/nacubactam against a neutropenic murine model of thigh infection with NDM-1-positive *K. pneumoniae* ATCC BAA-2473 (mean ± SD, *n* = 3). Aztreonam was administered every 2 h, and nacubactam was administered with variable frequency: every 2 h, every 4 h and every 8 h.

An *in vivo* dose-fractionation study in neutropenic mice thigh-infected with *K. pneumoniae* ATCC^®^ BAA-2473 showed that aztreonam and nacubactam alone hardly reduced bacterial counts. However, the aztreonam/nacubactam combination showed a nacubactam dose-dependent bacterial reduction and a strong bactericidal effect of up to 2 log_10_ cfu/thigh compared with the control group (Figure 4).

**Figure 5.**
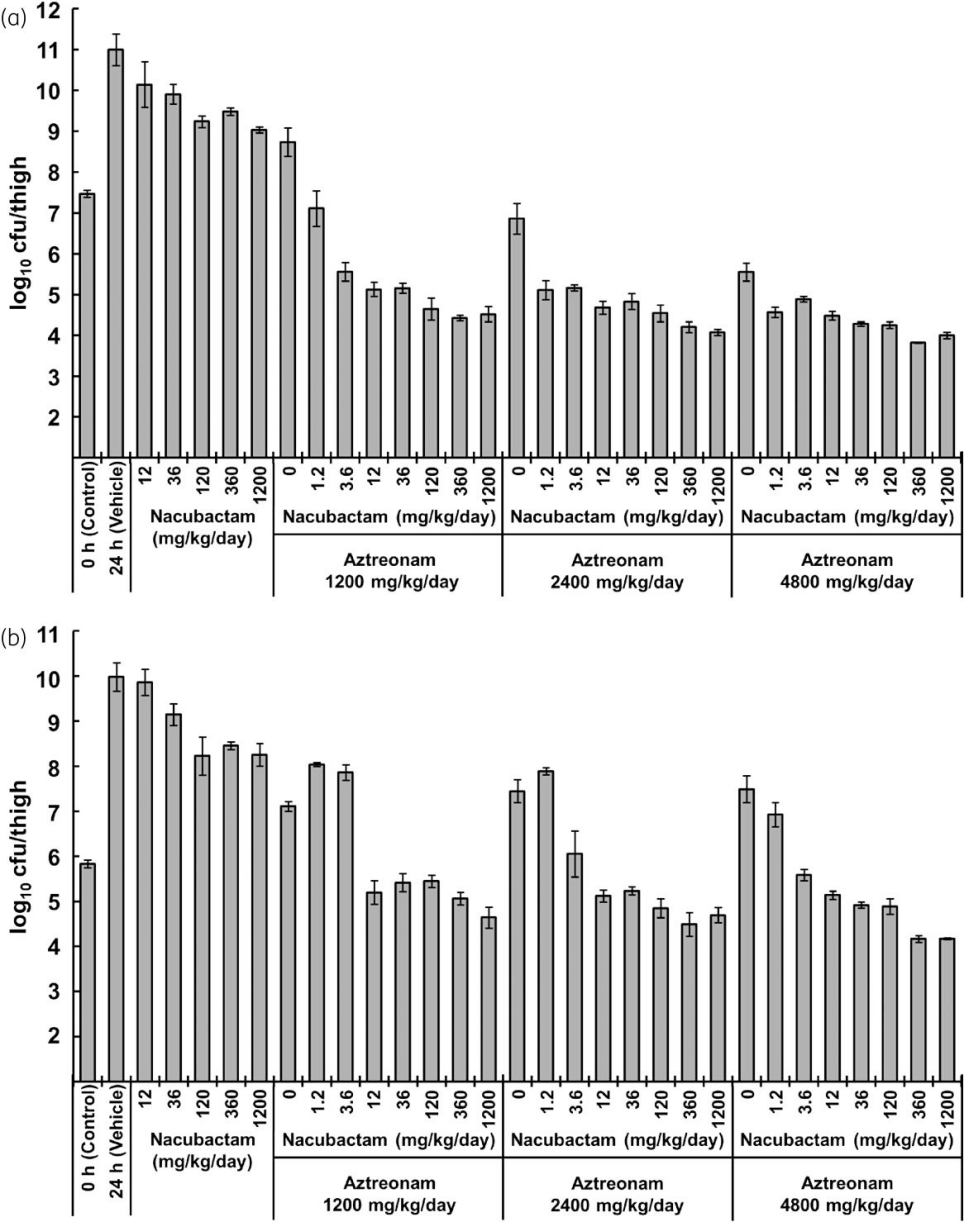
*In vivo* dose-ranging study of aztreonam/nacubactam against a neutropenic murine model of thigh infection with IMP-6-positive *K. pneumoniae* MSC 21664 (a) and OXA-48-positive *K. pneumoniae* MSC 21444 (b) (mean ± SD, *n* = 3). Both nacubactam and aztreonam were administered every 2 h.

The *in vivo* dose-ranging study of aztreonam/nacubactam was assessed in neutropenic thigh-infected mice with *K. pneumoniae* MSC 21664 and MSC 21444, administering aztreonam/nacubactam at various doses q2h (Figure 5). In both strains, nacubactam and aztreonam alone showed a slight bacterial reduction, but the combination of both antibiotics provided a potent bactericidal effect. The maximum bactericidal effect in mice infected with *K. pneumoniae* MSC 21664 and MSC 21444 was about 3 log_10_ and 1 log_10_ cfu/thigh, respectively.

### PK/PD analysis with fT_>MICi_

**Figure 6.**
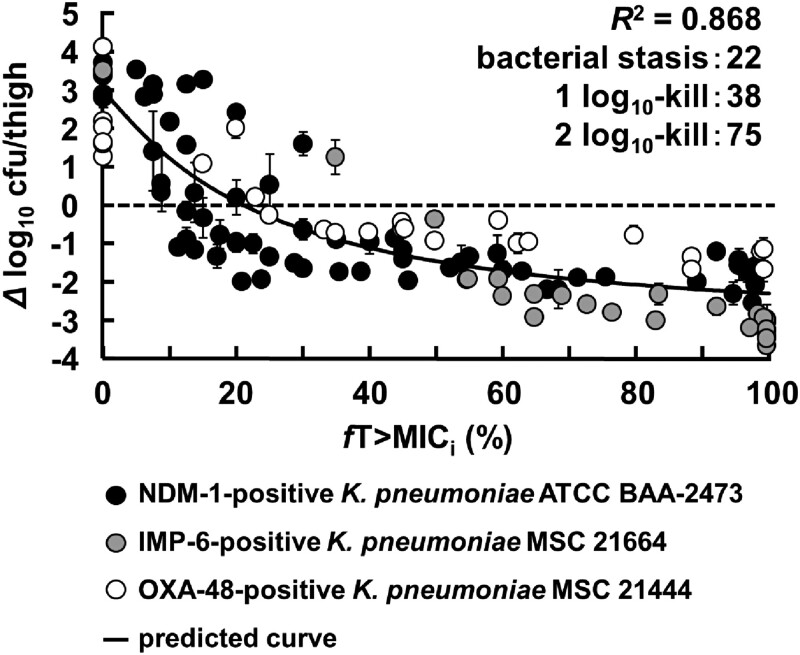
PK/PD analysis and target *fT*_>MICi_ values of aztreonam with/without nacubactam.

The values of *fT*_>MICi_ in each dose group calculated from the PK data were plotted against the change in bacterial counts obtained in the PD study (Figure 6). The results showed a high correlation (*R*^2 ^= 0.868) between the change in viable bacterial counts and *fT*_>MICi_ following aztreonam/nacubactam administration. The growth inhibition and bactericidal effect target values were further analysed by Equation 2. The target values of *fT*_>MICi_ required to achieve growth inhibition, 1 log_10_ kill and 2 log_10_ kill were 22%, 38% and 75%, respectively.

### PK/PD analysis with C_T_

**Figure 7.**
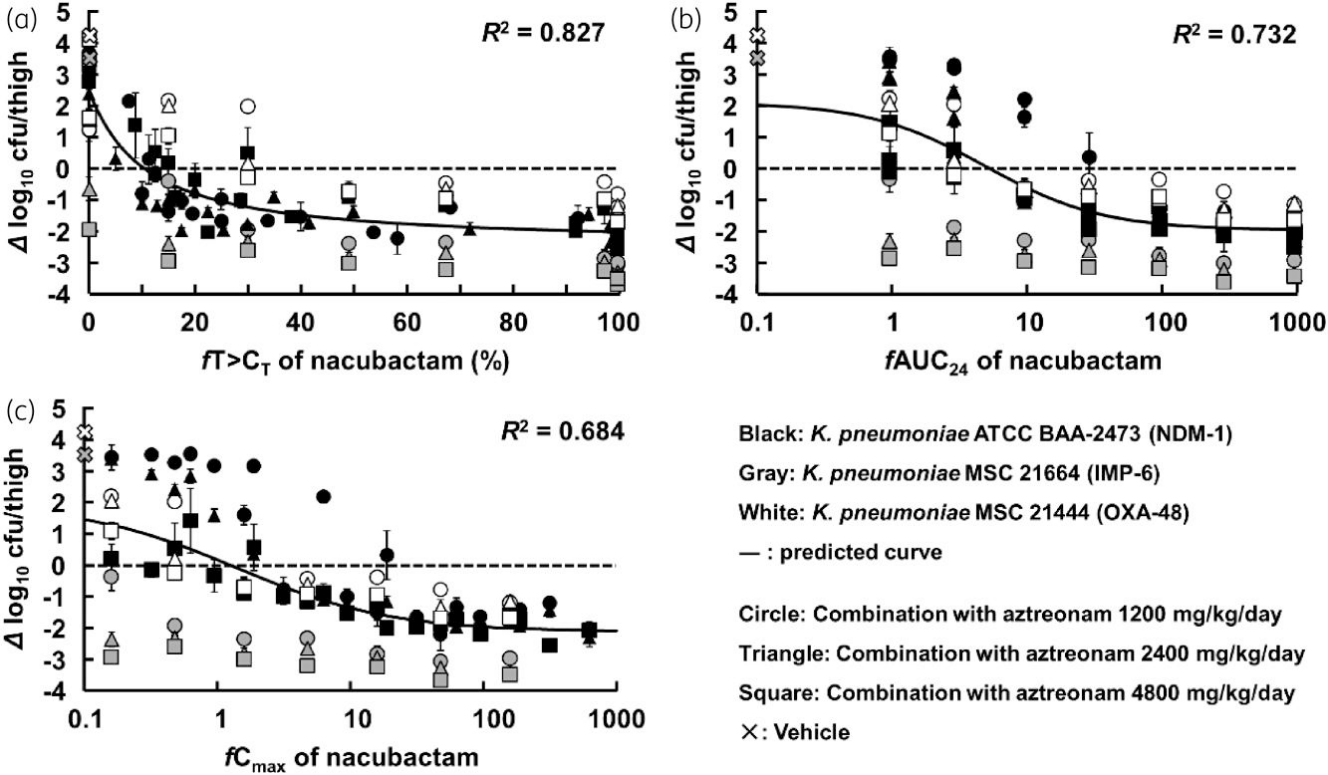
PK/PD analysis based on *fT*>*C*_T_ with optimal *C*_T_ values of nacubactam (a), *f*AUC_24_ of nacubactam (b), and *fC*_max_ of nacubactam (c).

The values of *fT*>*C*_T_ in each dose group calculated from the PK data were plotted against the change in bacterial counts obtained in the PD study. The *R*^2^ value in each *C*_T_ of nacubactam was analysed by Equation 2 (Figures S3–S5). Figures S3–S5 summarize the relationship between *C*_T_ of nacubactam and *R*^2^ values. The *C*_T_ values with the highest *R*^2^ value for *K. pneumoniae* ATCC^®^ BAA-2473 when combined with aztreonam 1200, 2400 and 4800 mg/kg/day were 2, 1 and 0.125 mg/L, respectively (Figure S3). For *K. pneumoniae* MSC 21664 and MSC 21444, the highest *R*^2^ values were obtained at *C*_T _= 0.125 mg/L for all doses (Figures S4 and S5). The optimum *C*_T_ value was set for each strain and the dose of aztreonam in combination, and an analysis was performed collectively, and the *R*^2^ value was 0.827 (Figure 7). Analysis of the relationship between the *f*AUC or *fC*_max_ of nacubactam and bacterial counts revealed that the *R*^2^ value was lower than *fT*>*C*_T_ (Figure 7). The *R*^2^ value for these parameters using only nacubactam exposure are inferior to those of the *fT*_>MICi_ analysis, meaning that an integrated PK/PD approach is needed.

## Discussion

PK/PD parameters of β-lactams/β-lactamase inhibitors are challenging to determine. This is because some β-lactamase inhibitors have antimicrobial activity in addition to protecting β-lactams from β-lactamases, and the antimicrobial effects of various combinations are interdependent. There is a need to establish accurate analytical methods to determine the PK/PD parameters of β-lactams/β-lactamase inhibitors because inappropriate analytical methods or models with low predictive accuracy risks creating a dissociation between susceptibility data *in vitro* and actual clinical efficacy. The current research shows how the MIC_i_ concept may be used in conventional PK/PD analysis to produce PK/PD parameters that are more practical, versatile and accurate.

The first-generation β-lactamase inhibitor combination piperacillin/tazobactam has been tested for the MIC of piperacillin in a fixed concentration of tazobactam of 4 mg/L, at a dose ratio of 8:1. On the other hand, amoxicillin/clavulanate is tested for susceptibility at a fixed 2:1 ratio, although several formulations with ratios of 2:1, 4:1 and 7:1 are used and may not correlate with efficacy. This *in vitro* and *in vivo* difference in combination ratios may be due to the fluctuating susceptibility of β-lactams in response to changing inhibitor concentrations over time being ignored during *in vivo* analysis. Optimization of *fT*>*C*_T_ assumes that the susceptibility of β-lactams is constant in the presence of β-lactamase inhibitors. However, MICs for clinical strains are not incorporated into the dosing design and do not adequately reflect changes in β-lactam susceptibility caused by different β-lactamase inhibitor concentrations.

In the present study, the MIC curve for aztreonam varied significantly in the therapeutic concentration range of nacubactam (approximately 0.1–10 mg/L) (Figure 2). In evaluating the PK/PD properties of β-lactams/β-lactamase inhibitors, this result supports the importance of considering changes in sensitivity under different β-lactamase inhibitor concentration conditions. On the other hand, *fT*_>MICi_ can reflect changes in β-lactam susceptibility and changes in β-lactamase inhibitor concentration. Comparing the PK/PD analysis results from the two approaches, the *R*^2^ values were higher for *fT*_>MICi_ than for *fT*>*C*_T_, although curves of similar shape were drawn (Figures 6 and 7). The low *R*^2^ value for *fT*>*C*_T_ may be due to underestimating the PK/PD predictions based on *fT*>*C*_T_. Higher aztreonam concentrations are clinically effective even when nacubactam concentrations are below the *C*_T_ value. Therefore, *fT*_>MICi_ is considered a more useful PK/PD parameter than *fT*>*C*_T_.

The antibacterial activity of interdependent concomitant drugs is affected by factors such as bacterial species, enzyme type, enzyme expression level, antibacterial activity and inhibitory activity, and concentration-dependent additive/synergistic effects. However, in the clinical treatment of infectious diseases, the data available for designing the administration of antibiotics are limited. In addition, carbapenemase-producing clinical isolates that co-produce multiple β-lactamases are not uncommon in practice, and there is little significance in setting PK/PD targets for each strain. Therefore, it would be desirable to define a single PK/PD target that can accommodate a wide variety of clinical isolates, allowing optimal dosing design based on limited MIC data. PK/PD analysis with *fT*>*C*_T_ confirmed that the *C*_T_ value of nacubactam differed depending on the strain and the dose of concomitant aztreonam (Figures S3–S5). Therefore, it lacks versatility according to individual patients, such as strains that produce multiple types of β-lactamases and patients with renal dysfunction that affects pharmacokinetics. On the other hand, it is worth noting that dosing design based on *fT*_>MICi_ does not need to consider these factors and therefore has good practical versatility. The chequerboard MIC curves reflect enzyme type, inhibitory activity, antimicrobial activity, and the additive and synergistic effects of antimicrobial agents. By simulating based on these chequerboard MIC curves, it is considered that a single PK/PD target value can be obtained even with different genotypes and different inhibitory activities. Indeed, the analysis by *fT*_>MICi_ showed a high correlation when analysed for each strain (Figure S6), but a good correlation (*R*^2 ^= 0.868) was also obtained when the results of three β-lactamase-producing strains with different genotypes were analysed together (Figure 6).

The novel DBO-based β-lactamase inhibitor, nacubactam, is a potent inhibitor of Ambler class A (e.g. KPC and ESBL) and C (AmpC) β-lactamases but weakly inhibits class D (OXA) β-lactamases.^20,21^ In addition, nacubactam exhibits antibacterial activity through binding to the bacterial PBP-2. Furthermore, unlike first-generation β-lactamase inhibitors (e.g. sulbactam, clavulanate, tazobactam), second-generation DBO-based β-lactamase inhibitors, including nacubactam, do not have a β-lactam structure and are not subject to β-lactamase degradation. Therefore, they are expected to be a new treatment option for carbapenemase (MBL)-producing bacteria, including class B (NDM and IMP),^22,23^ because no other established treatment is available. In the current chequerboard assay, aztreonam/nacubactam showed good combination efficacy against class B and D β-lactamase-producing bacteria such as NDM-1, IMP-6 and OXA-48 β-lactamase-producing *K. pneumoniae* (Table 1). Furthermore, the MIC of nacubactam against all bacterial species in the presence of 4 mg/L aztreonam reflects the minimum nacubactam concentration to achieve the microbiological breakpoint,^16^ and can be easily achieved in clinical practice, indicating aztreonam/nacubactam *in vivo* potent antimicrobial effect is obtainable. Furthermore, aztreonam/nacubactam combination therapy was effective in the murine model against NDM-1-, IMP-6- and OXA-48-producing *K. pneumoniae* in a nacubactam dose-dependent manner, despite aztreonam monotherapy being ineffective. Thus, the present study provides the first evidence of a satisfactory therapeutic effect of nacubactam against refractory β-lactamase-producing *K. pneumoniae*, particularly the *in vivo* antibacterial effect of aztreonam/nacubactam against NDM-1-producing bacteria.

There are two drawbacks to this research. The first is that all PD experiments only measured activity against fixed inoculum levels (approximately 10^6^ cfu/mL). The effect of greater inoculum levels on aztreonam/nacubactam drug efficacy is unknown. Due to the inoculum effect, several antimicrobials have lower antimicrobial effectiveness at higher initial inoculum levels.^24^ Some β-lactam/β-lactamase inhibitors, on the other hand, have been less influenced by the inoculum effect. Ceftazidime/tazobactam, for example, showed decreased action at high bacterial levels, but ceftazidime/avibactam was said to be unaffected by inoculum levels.^14^ The causes for this are unknown. However, the inoculum effect could be related to avibactam’s reversible inhibitory action and resistance to enzymatic hydrolysis. Because nacubactam inhibits in the same way that avibactam does, it may be less vulnerable to the inoculum effect. On this point, more evidence is required. Second, the site of action of the antimicrobials was the femoral tissue, but plasma drug concentrations were used for PK. Although evaluation of drug concentration at the infection site can improve the accuracy of predicting therapeutic efficacy, quantification of the drug in the interstitial fluid of murine thigh tissue is difficult. Therefore, PK/PD studies using murine thigh-infection models are usually analysed based on free drug blood concentrations, assuming equal free drug concentrations in blood and thigh tissue. Further studies using models capable of assessing drug concentrations at the infection site, such as pneumonia models, are needed to obtain a more accurate exposure–response relationship. Finally, adopting MIC_i_, a comprehensive concept, a new PK/PD analysis approach for β-lactam/β-lactamase inhibitor combination therapy was developed. In comparison with existing PK/PD parameters (*fT*>*C*_T_), *fT*_>MICi_ and their target values in this newly designed PK/PD analysis approach were more practical, generic and accurate.

## Supplementary Material

dkad033_Supplementary_Data

## Supplementary data

Figures S1 to S6 and Tables S1 to S3 are available as Supplementary data at *JAC* Online.
