# Supplementary material for: Development of an optimized and practical pharmacokinetics/pharmacodynamics analysis method for aztreonam/nacubactam against carbapenemase-producing K. pneumoniae
Source: J Antimicrob Chemother. 2023 Feb 13;78(4):991–9. doi: 10.1093/jac/dkad033 (PMC10068424; doi:10.1093/jac/dkad033)
Supplement: dkad033_Supplementary_Data [file dkad033_supplementary_data.docx]

**Fig. S1 Administration plan of dose ranging study and dose fractionation study images. (A) Dose ranging study: aztr
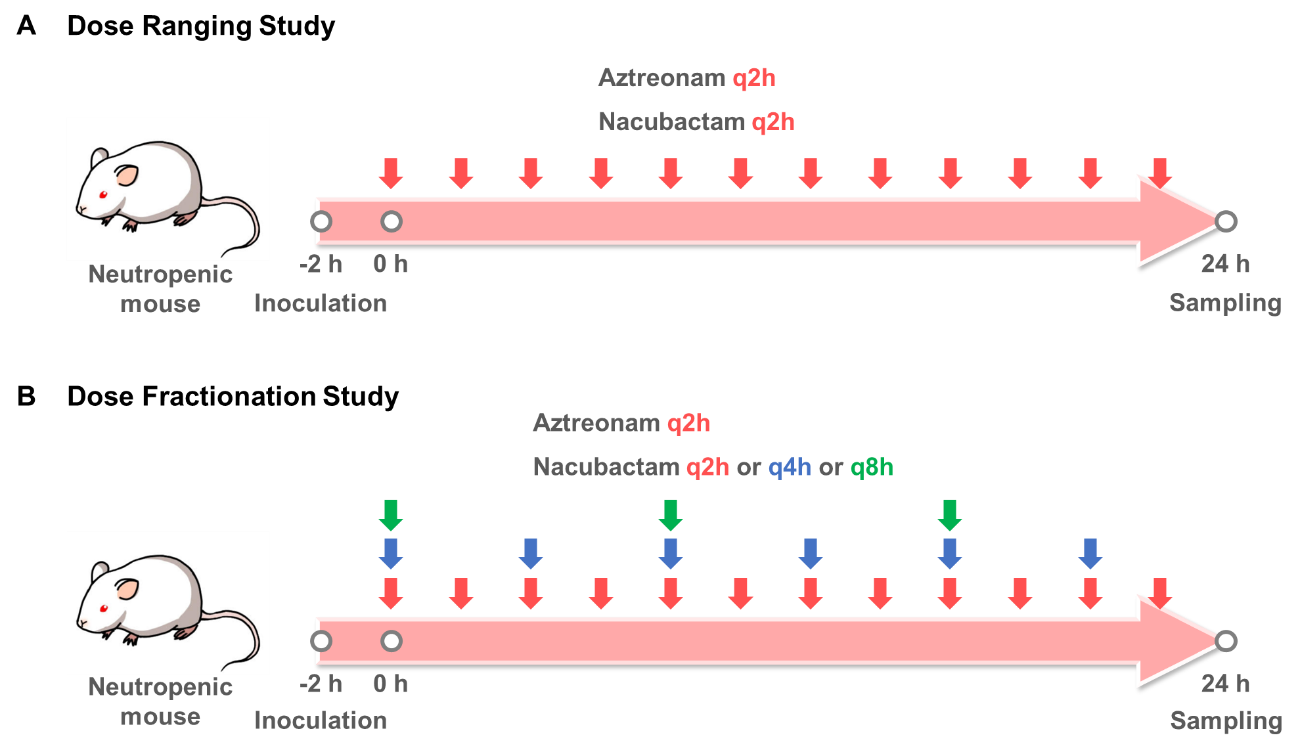
eonam (0, 1,200, 2,400, 4,800 mg/kg/day) and nacubactam (0, 1.2, 3.6, 12, 36, 120, 360, 1,200 mg/kg/day) were administered every 2 hrs. (B) Dose fractionation study: aztreonam (0, 1,200, 2,400, 4,800 mg/kg/day) was administered every 2 hrs and nacubactam (0, 1.2, 3.6, 12, 36, 120, 360, 1,200 mg/kg/day) was administered every 2 or 4 or 8 hrs.**

**
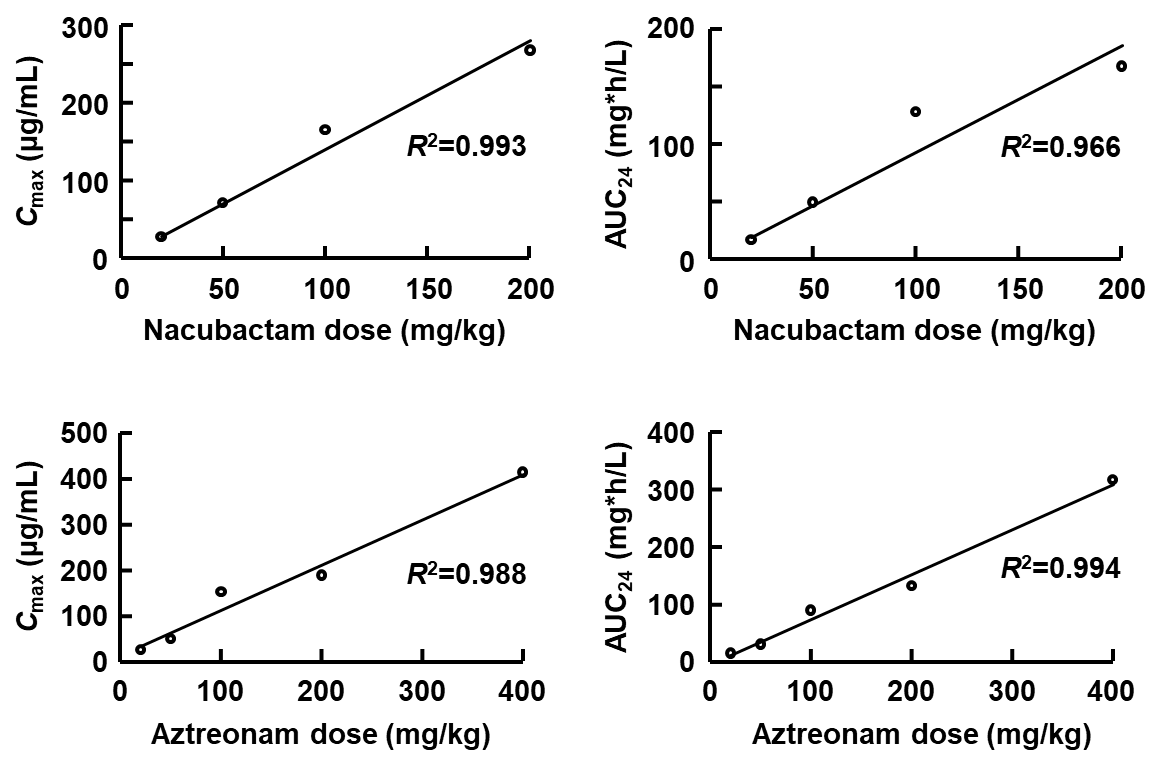
Fig. S2 *C*_max_ and AUC_24_ for nacubactam doses or aztreonam doses**

**Table S1 Method for measuring plasma drug concentrations using LC-MS/MS**

|  | **Nacubactam** | **Aztreonam** |
| --- | --- | --- |
| **Sample Preparation** | 50 µL of samples were subjected to the following pretreatments   - 50 µL of 50% acetonitrile was added - 30 µL of cephalexin solution (10 µg/mL) was added - 100 µL of methanol and acetonitrile was added - Centrifuged (10,000 rpm, 4°C, 5 min) - The supernatant was diluted ×2 with ultrapure water | |
| **LC system** | Acquity UPLC^®^ BEH, 1.7 µm, 2.1 mm×50 mm column (Waters) was used for analysis. 5 µL of the pretreated sample mixture was injected. The LC system consisted of mobile phase A (5 mM dibutyl ammonium acetate solution) and mobile phase B (0.1% formic acid in acetonitrile) in a gradient at 40°C. Analytes were eluted at 0.1 mL/min. | |
| **MS/MS** | API3200 (AB SCIEX, Tokyo, Japan) | |
| **Ionization mode** | ESI negative | |
| **Multiple reaction monitoring** | 323>96 | 434>96 |
| **Collision energy** | 40 eV | 24 eV |
| **Quantitative range** | 10 to 2,500 ng/mL | |

**Table S2 PK parameters of nacubactam and aztreonam in neutropenic mice (n=3).**

| Antimicrobial | *k*_a_  (h^-1^) | *k*_e_  (h^-1^) | *k*_12_  (h^-1^) | *k*_21_  (h^-1^) | *V*_1_/*F*  (L/kg) | *V*_2_/*F*  (L/kg) | | CL_tot_/*F*  (L/h/kg) | *t*_1/2_  (h) |
| --- | --- | --- | --- | --- | --- | --- | --- | --- | --- |
| Nacubactam | 7.35  (3.64) | 4.27  (1.02) | 0.28  (0.11) | 0.39  (0.28) | 0.29  (0.10) | 0.49  (0.58) | 1.14  (0.13) | | 0.18  (0.05) |
| Aztreonam | 6.00  (2.61) | 4.77  (3.04) | 0.05  (0.04) | 0.25  (0.30) | 0.40  (0.21) | 0.43  (0.54) | 1.35  (0.18) | | 0.20  (0.10) |

Data are presented as means (S.D.).

Abbreviation: *k*_a_, absorption rate constant; *k*_e_, disappearance rate constant; *k*_12_, distribution rate constants from the central into the peripheral compartment; *k*_21_, distribution rate constants from the peripheral into the central compartment; *V*_1_/*F*, volume of distribution of the central compartment; *V*_2_/*F*, volume of distribution of the peripheral compartment; CL_tot_/*F*, total clearance; *t*_1/2_, half-life.

**Table S3 Protein binding rate of nacubactam and aztreonam in mouse plasma**

| **Antimicrobial** | **Concentration** | **Mean (n=3)** | **S.D.** | **Mean** |
| --- | --- | --- | --- | --- |
| **Nacubactam** | 400 mg/L | 5.20 | 5.02 | 3.87 |
|  | 10 mg/L | 0.91 | 3.91 |  |
|  | 1 mg/L | 5.51 | 13.13 |  |
| **Aztreonam** | 400 mg/L | 45.80 | 3.96 | 57.09 |
|  | 10 mg/L | 65.01 | 4.09 |  |
|  | 1 mg/L | 60.45 | 4.32 |  |

**
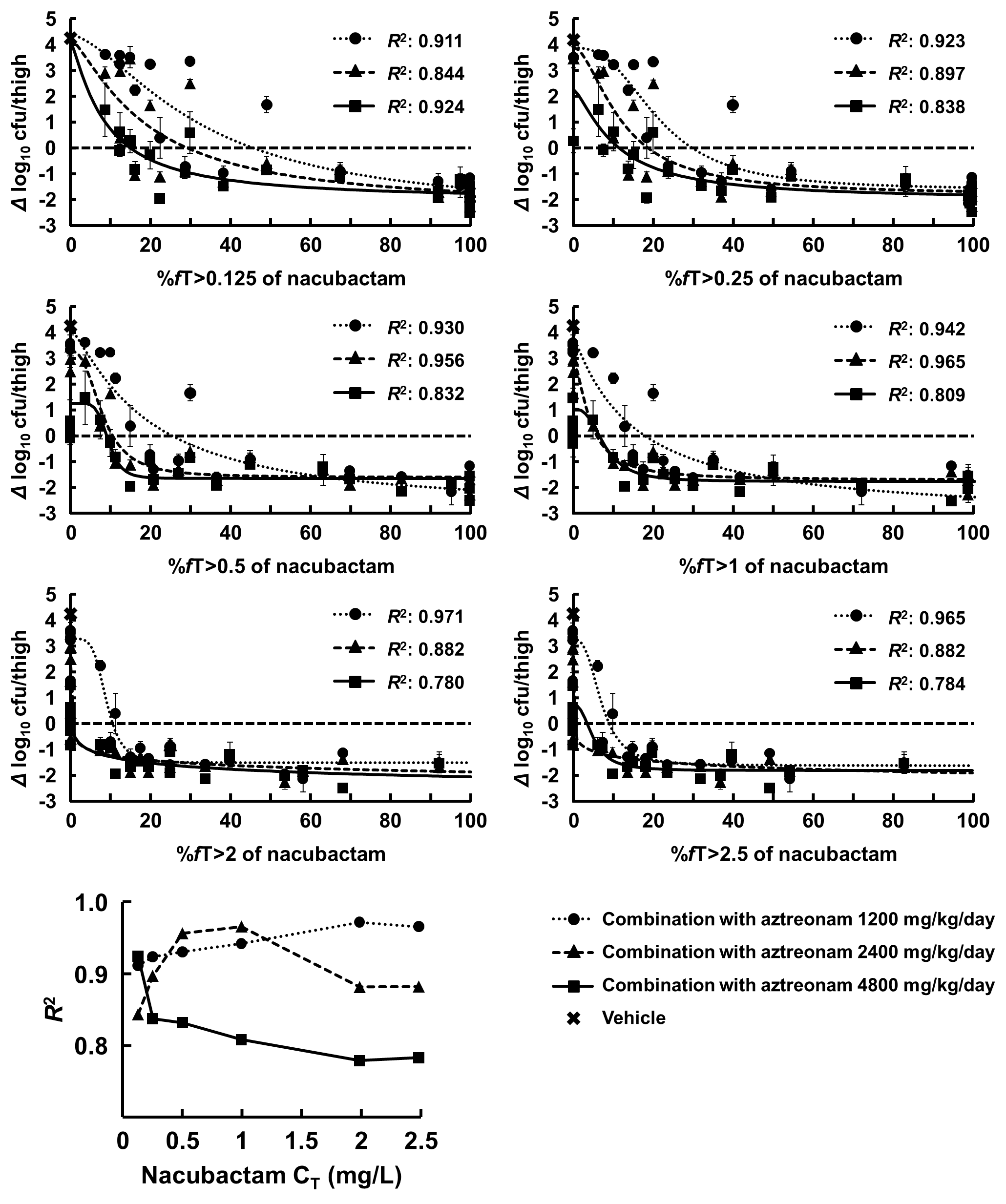
**

**Fig. S3 Relationship of %*f*T>C_T_ of nacubactam and the change in the log_10_ cfu/thigh of NDM-1-positive *K. pneumoniae* ATCC BAA-2473.**

**
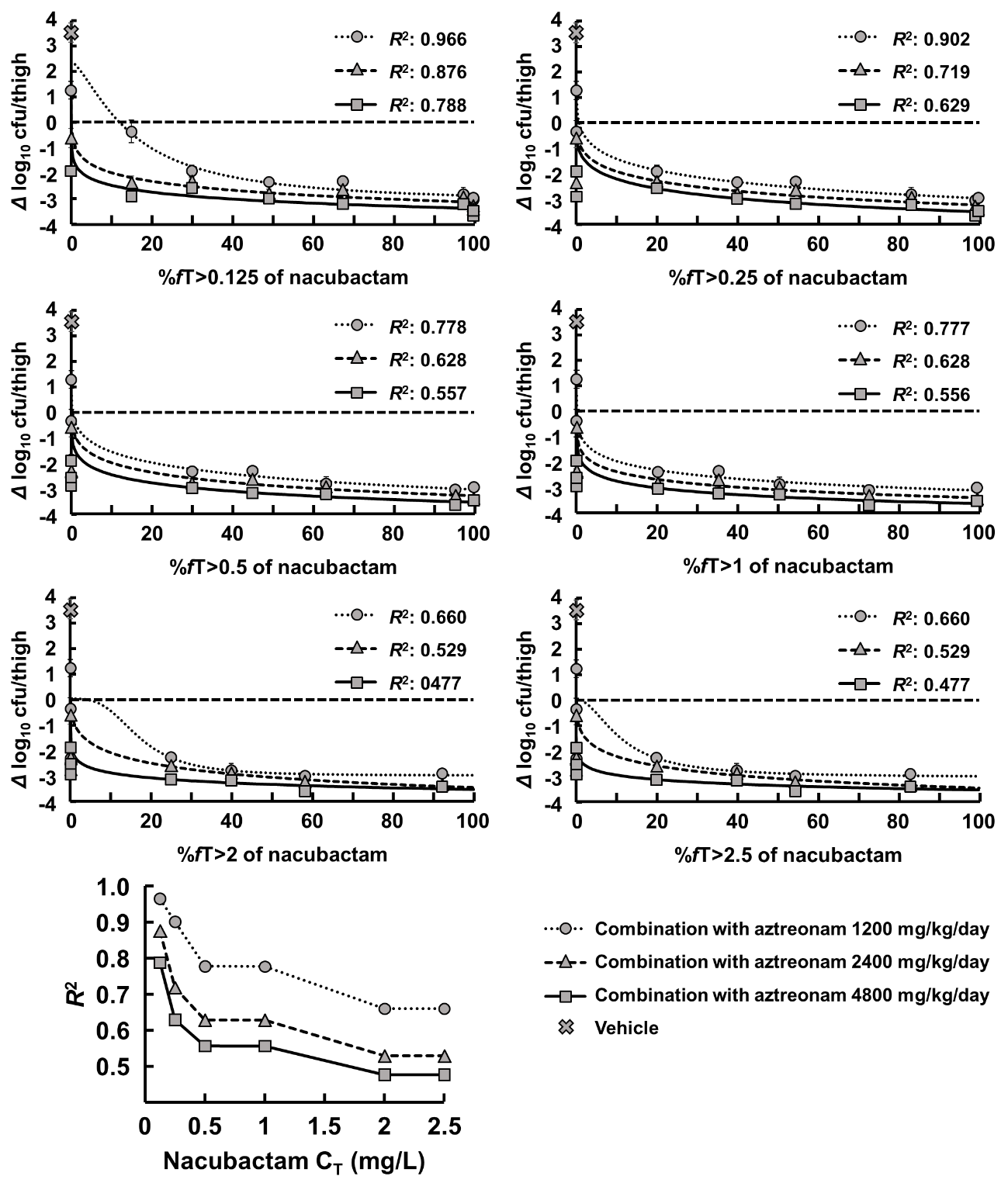
Fig. S4 Relationship of %*f*T>C_T_ of nacubactam and the change in the log_10_ cfu/thigh of IMP-6-positive *K. pneumoniae* MSC 21664.**

**
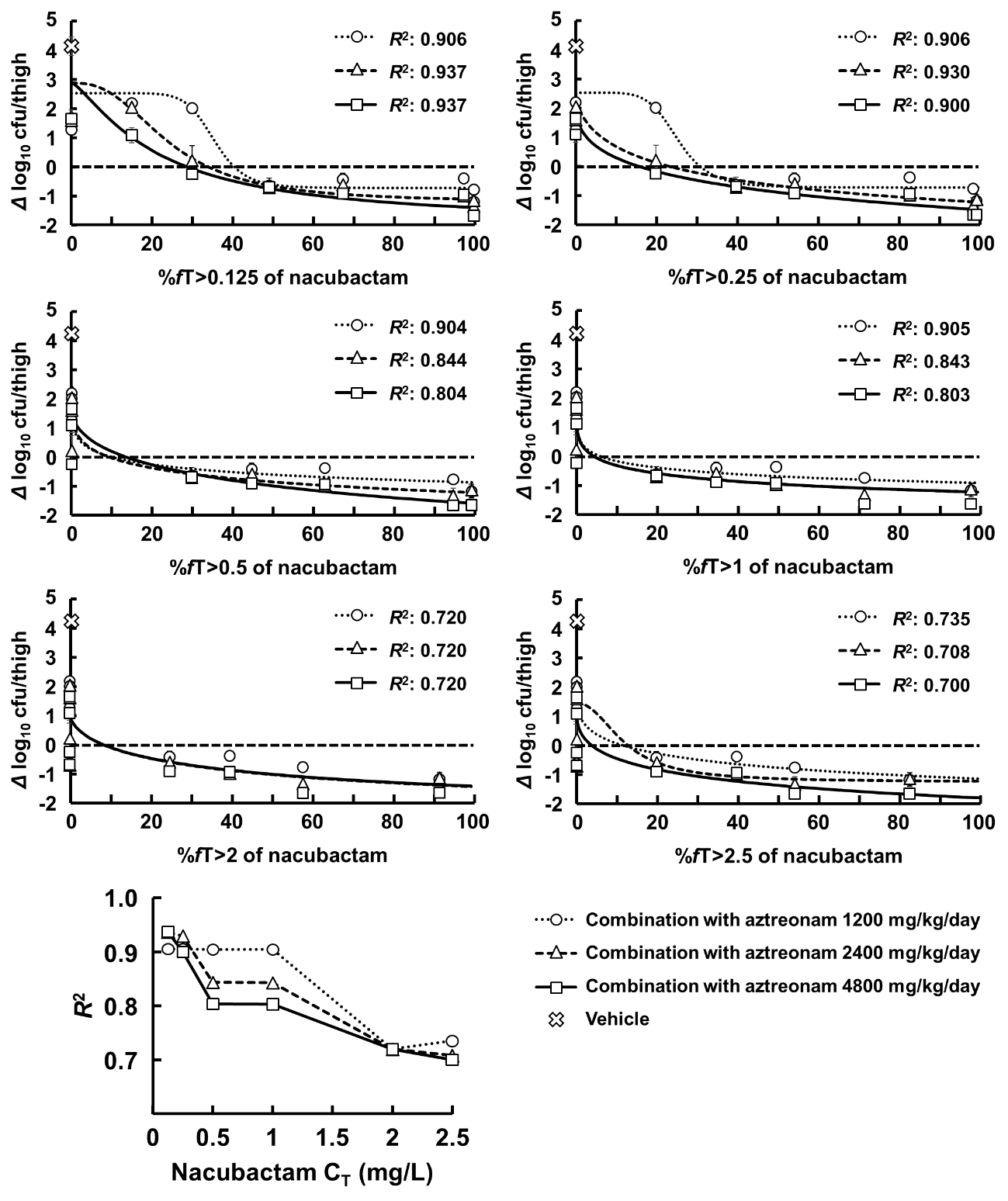
Fig. S5 Relationship of %*f*T>C_T_ of nacubactam and the change in the log_10_ cfu/thigh of OXA-48-positive *K. pneumoniae* MSC 21444.**

**
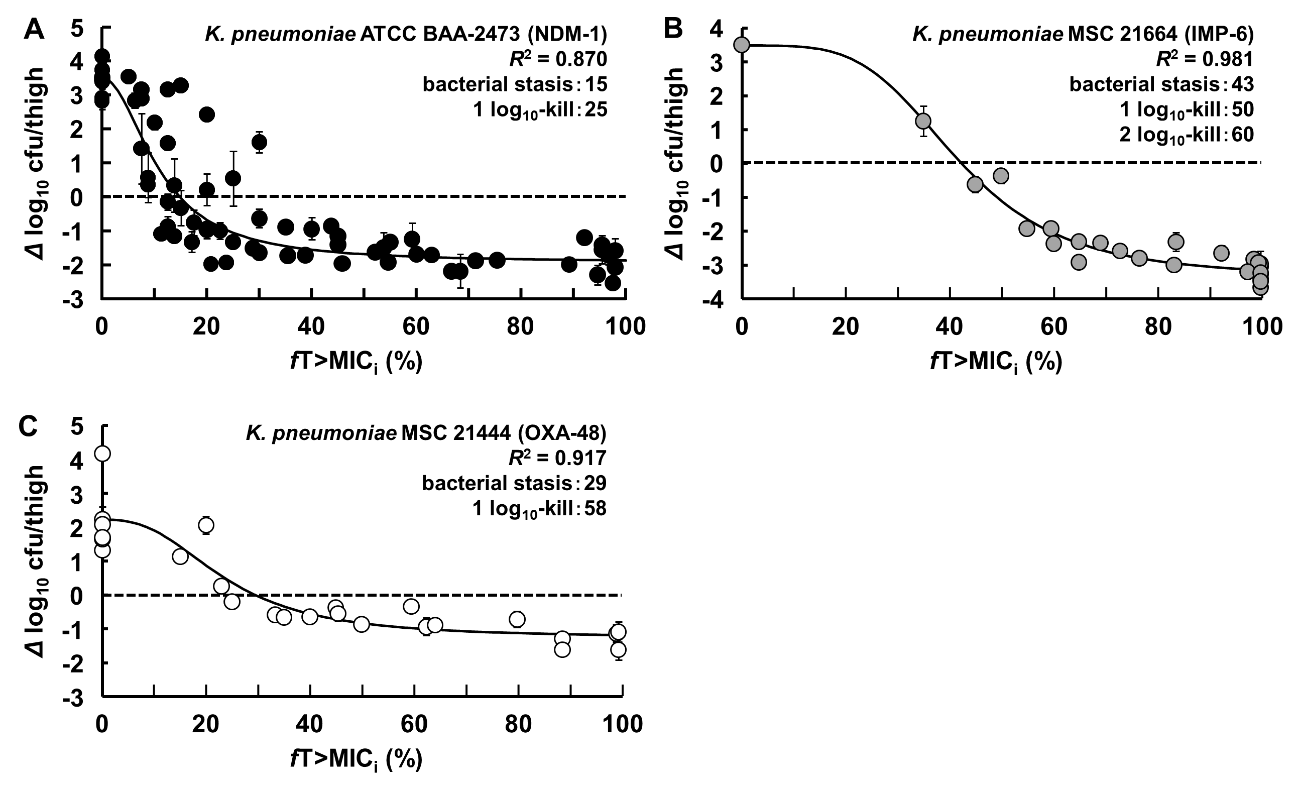
Fig. S6 PK/PD analysis and target *f*T>MIC_i_ values of aztreonam/nacubactam against *K. pneumoniae* ATCC BAA-2473 (A), MSC 21664 (B) and MSC 21444 (C).**
